# A compartmentalized microfluidic neuromuscular co-culture system reveals spatial aspects of GDNF functions

**DOI:** 10.1242/jcs.167544

**Published:** 2015-03-15

**Authors:** Eitan Erez Zahavi, Ariel Ionescu, Shani Gluska, Tal Gradus, Keren Ben-Yaakov, Eran Perlson

**Affiliations:** Department of Physiology and Pharmacology, Sackler Faculty of Medicine, and the Sagol School of Neuroscience, Tel Aviv University, Tel Aviv 69978, Israel

**Keywords:** Axon degeneration, Axonal transport, GDNF, Microfluidic chamber, Neuromuscular junction, Neurotrophic factors

## Abstract

Bidirectional molecular communication between the motoneuron and the muscle is vital for neuromuscular junction (NMJ) formation and maintenance. The molecular mechanisms underlying such communication are of keen interest and could provide new targets for intervention in motoneuron disease. Here, we developed a microfluidic platform with motoneuron cell bodies on one side and muscle cells on the other, connected by motor axons extending through microgrooves to form functional NMJs. Using this system, we were able to differentiate between the proximal and distal effects of oxidative stress and glial-derived neurotrophic factor (GDNF), demonstrating a dying-back degeneration and retrograde transmission of pro-survival signaling, respectively. Furthermore, we show that GDNF acts differently on motoneuron axons versus soma, promoting axonal growth and innervation only when applied locally to axons. Finally, we track for the first time the retrograde transport of secreted GDNF from muscle to neuron. Thus, our data suggests spatially distinct effects of GDNF – facilitating growth and muscle innervation at axon terminals and survival pathways in the soma.

## INTRODUCTION

Motoneurons extend axons over long distances and through varying extracellular microenvironments to form synapses with muscles. The formation and maintenance of these neuromuscular junctions (NMJs) depend on both internal and external signals that need to integrate with specificity and fidelity over space and time (Shi et al., 2012; Wu et al., 2010). Studies on NMJs have shed light on some central signaling pathways and principles of synapse assembly (Shi et al., 2012; Sanes and Lichtman, 1999). Still, little is known of the mechanism of retrograde and anterograde signaling between the neuron and the muscle. Much of the difficulty in deciphering these mechanisms is due to the technical challenges of studying these complex intra- and extracellular communications at the subcellular level.

*In vitro* compartmental systems that separate neuronal cell bodies from their axons and synapses are becoming a progressively useful tool for researchers (Taylor et al., 2005). Such platforms enable the precise control, monitoring and manipulation of cellular microenvironments. Motoneurons have been adapted to culture conditions in microfluidic chambers (Eleftheriadou et al., 2014; Restani et al., 2012), but so far this type of platform has not been widely used for studying the mechanisms of NMJ formation and maintenance, although recent publications describe initial efforts in this direction (Park et al., 2013; Southam et al., 2013).

Motoneuron–muscle communication is vital for NMJ formation and maintenance, as well as for motoneuron survival and proper function. Alterations in such intercellular communication can lead to synapse disruption and axon degeneration, and might be a crucial step in neurodegenerative diseases. The bidirectional communication process is conducted by both adherent and secreted factors, and mediated by ligand–receptor mechanisms. These signals are thought to either act locally or be conveyed by long-distance retrograde axonal transport. GDNF is one such signaling factor that plays a role in motoneuron survival and facilitates NMJ maturation (Kanning et al., 2010; Paratcha and Ledda, 2008). GDNF is a secreted factor from the TGFβ superfamily and is one of the most potent motoneuron survival factors characterized to date (Henderson et al., 1994; Oppenheim et al., 1995). The expression of the GDNF receptors and co-receptors RET tyrosine kinase, neuronal cell adhesion molecule (NCAM) and GDNF family receptor alpha-1 (GFRA1) at the NMJ (Sariola and Saarma, 2003) suggests that GDNF might be taken up and internalized at the NMJ (Houenou et al., 1996). GDNF is thought to be a muscle-derived factor that regulates NMJ formation and maintenance. However, to date, no studies have directly tracked the uptake of GDNF by motoneurons and its transport along the axon. Moreover, the molecular mechanisms of GDNF effects in the development and function of NMJs are not well understood, and a direct demonstration of spatially specific roles of GDNF is still lacking (Coulpier and Ibáñez, 2004; Tsui and Pierchala, 2010).

Here, we investigate mechanisms of motoneuron–muscle communication and NMJ maintenance using an *in vitro* microfluidic platform with motoneuron cell bodies on one side and primary muscle cells on the other. First, we demonstrate the formation of pre- and post-synaptic domains of functional NMJs in the compartmental co-culture. Then, we observed higher vulnerability of the NMJ compartment to oxidative stress. Next, we confirmed a dual spatial role of GDNF. GDNF applied at the NMJ but not in the soma compartment facilitates axon growth and formation of active neuromuscular synapses. Finally, we tracked the retrograde transport of GDNF from muscle to neuron, demonstrating that a factor secreted by muscle mediates retrograde communication with motoneurons, leading to the activation of the survival pathway.

## RESULTS

### Co-culture of muscle and motoneurons in a microfluidic chamber

**Fig. 1.**
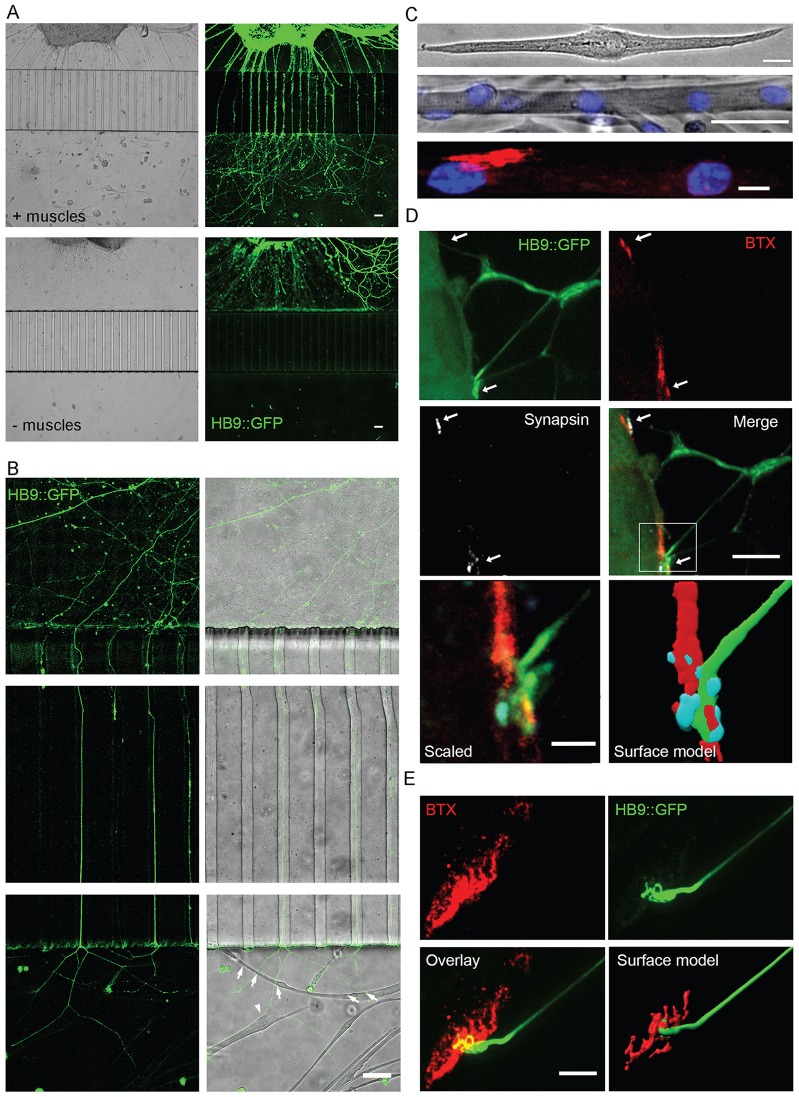
**Axonal growth and innervation of differentiated myotubes in the MFC distal compartment.** (A) Epifluorescent and corresponding brightfield images at low magnification (4× objective) of spinal cord explants growing HB9::GFP-expressing (green) axons across the microgrooves in response to muscles grown in the distal compartment (upper panels), in comparison to explants cultured without muscles (lower panels). (B) Close-up (20× objective) epifluorescent images of HB9::GFP-expressing axons growing in the proximal compartment, crossing the microgrooves and innervating myotubes in the distal compartment (upper, middle and lower panels, respectively). Arrows and arrowheads denote poly and mono-innervated myotubes, respectively. (C) Cultured myoblasts differentiate into elongated (upper image) and polynuclear striated myotubes, which were stained with Hoecsht 33258 (blue) and imaged by using a 20× objective in the Floid imaging station (middle image). Elongated polynuclear myotubes display large AChR clusters (labeled by BTX-594, red) independent of innervation (lower image). (D) High-magnification images taken with Lecia SP8 confocal microscope with a 63× objective showing formation of clusters with HB9::GFP (green), synapsin (white) and post-synaptic AChR (BTX, red) NMJ markers in axon–muscle contact areas. The arrows indicate sites of pre- and post-synaptic marker colocalization on the myotube surface. The lower panel shows a zoom-in of the contact area (left image) and a 3D surface model (right). (E) Confocal image stack projection of stained co-cultures showing HB9::GFP-expressing axons reaching an AChR pretzel-shaped cluster on a myotube. The lower-right image shows a 3D surface model of the neuromuscular contact area. See also supplementary material Movie 1. Scale bars: 50 µm (A; B; C, middle panel), 20 µm (C, upper panel), 5 µm (C, lower panel; D, upper panels; E), 1 µm (D, lower panel).

To study spatial and temporal processes occurring at the NMJ sites compared to the cell body site, and the bidirectional communication between neurons and muscle, we used the compartmental microfluidic system (Park et al., 2006) to create an optimized compartmental motoneuron–muscle platform. The microfluidic chamber (MFC) consists of two main compartments or channels that are connected by parallel grooves. A 0.5–1-mm piece of the ventral horn of spinal cord explant from an HB9::GFP mouse embryo is plated in the explant compartment, and myoblasts derived from primary satellite cells of the gastrocnemius muscle of an adult mouse are plated in the muscle compartment (supplementary material Fig. S1A). Myoblasts fuse and differentiate spontaneously into skeletal myotubes at 1–3 days after plating in the chamber; simultaneously, HB9::GFP motoneurons grow their processes through the microgrooves and into the muscle compartment over a similar period of ∼3 days (Fig. 1A). Thus, this co-culture system separates muscle and neuron somata, while allowing axons to grow and innervate the muscle in the distal compartment. Interestingly, when growing HB9::GFP explants in the absence of muscle cells, we observed slower axon growth through the grooves, whereas neurite growth in the proximal neuronal compartment was similar under both conditions (Fig. 1A). Hence, the presence of muscle cells in the distal compartment facilitates the extension of axons across the microgrooves, suggesting that the cultured myocytes secrete factors that attract axons and promote their growth. After crossing into the muscle side, HB9::GFP axons contact the differentiated myotubes directly, forming both poly- and mono-innervations of single myotubes (Fig. 1B). High-magnification images of the neuromuscular contact areas show formation of differentiated polynuclear muscle cells (Fig. 1C), as well as typical pretzel-like clusters of acetylcholine receptors (AChRs), and the presence of pre- and post-synaptic markers in those areas (Fig. 1D,E; supplementary material Movie 1).

### Identification and visualization of synapses within the microfluidic chamber

**Fig. 2.**
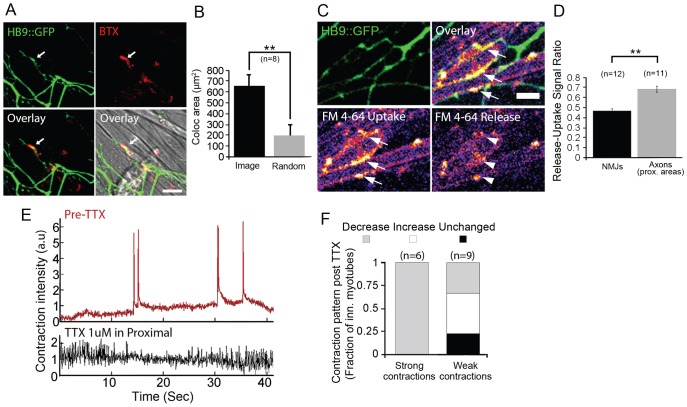
**Formation of functional neuromuscular junctions in the muscle compartment.** (A) Confocal images of specific colocalization (arrows) of AChR clusters in myotubes (BTX, red) and HB9::GFP (green) axons. Scale bar: 20 µm. (B) Mean colocalized area compared to random colocalization after image flipping (random). Eight images taken from a representative co-culture were analyzed. Data show the mean±s.e.m.; **P<0.01 (one-tailed Student's *t*-test). (C,D) Pre-synaptic uptake and release of FM-4-64 dye in NMJs. Representative confocal images using a 20× objective (left) showing HB9::GFP axon terminals (green) in contact with myotubes with puncta of uptaken FM dye (arrows) and 5 min after KCl-glutamate-induced release (arrowheads). Scale bar: 10 µm. The graph shows the mean fold reduction in the FM dye signal in NMJs compared to that in proximal (prox.) areas in a representative co-culture (±s.e.m.); **P<0.01 (one-tailed Student's *t*-test). (E) Representative time trace of contraction in an innervated myotube pre- and post-addition of 1 µM TTX to the neuronal compartment (see also supplementary material Movie 3). a.u., arbitrary units. (F) The fraction of myotubes displaying altered or unaffected levels of innervation-induced contractions compared to weak spontaneous contractions in innervated (inn.) myotubes post- and pre-TTX addition to the neuronal compartment.

To test whether synapses between motoneurons and myotubes can form within the muscle compartment, we analyzed the colocalization of HB9::GFP neurites and clusters of AChRs on the myotubes stained with bungarotoxin (BTX). Statistically significant colocalization of pre- and post-synaptic markers was found in the NMJ compartment (Fig. 2A,B), as verified in the same images in which the BTX channel was flipped against the GFP channel. These data suggest that motoneurons grow their processes toward clusters of AChRs in a directed manner. To show that innervating motoneuron axons are capable of pre-synaptic activity, we performed a functional assay of uptake and release of FM-4-64 dye. We compared the intensity of FM dye puncta colocalized with HB9 axon terminals and with myotube areas after uptake of the FM dye to its release levels after stimulation with KCl+glutamate. We saw a 60% larger fold reduction in the level of FM dye signal in areas juxtaposed to the myotube compared with that observed at areas along the axon that are farther from NMJs (Fig. 2C,D), demonstrating the release capability of motoneuron pre-synapses in the system. Thus, we validate synapse functionality by tracking alterations in FM dye intensity upon uptake and its release in response to stimulation.

**Fig. 3.**
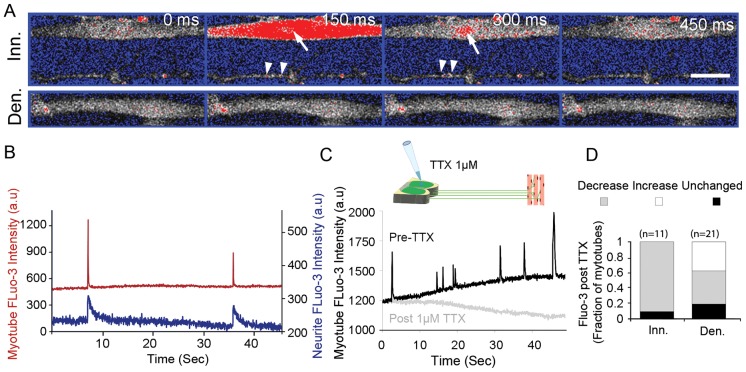
**Ca^2+^ imaging of myotubes and innervating axons.** (A) A correlated Fluo-3 transient in axon terminals and innervated (Inn.) myotubes. A representative timecourse of epifluorescent images was taken of a single spike (upper panel) with a 20× objective. Arrowheads and arrows mark signal spiking in the axon and myotube, respectively; lower images are of a non-innervated (Den.) myotube. Scale bar: 10 µm. (B) Representative time trace showing correlated Fluo-3 spiking in a neuron (blue) and an innervated myotube (red). a.u., arbitrary units. (C) Time trace of Fluo-3 signal in an innervated myotube pre- and post-TTX (1 µM) addition to the neuronal compartment. (D) The fraction of myotubes showing altered or unaffected levels of Fluo-3 signal spikes in innervated compared to denervated neurons after TTX treatment in the neuronal compartment.

To evaluate the functional significance of these innervations on muscle activity, we compared the contractile behavior of motoneuron-innervated and non-innervated myotubes. Regardless of innervation, differentiated muscles in the chamber showed spontaneous twitching. Innervated muscles, however, displayed irregular single or bursting contractions, which are much longer and stronger than the spontaneous twitching seen in the non-innervated muscles. The non-innervated muscles did not show this type of strong contraction. Thus, the ‘strong’ contractions might be induced by motoneurons stimulating innervated muscles. Further supporting this notion, we often observed these high-amplitude contractions occurring simultaneously in multiple non-connected myotubes innervated by the same axon (supplementary material Movie 2). Adding 1 µM tetrodotoxin (TTX), a blocker of voltage-gated sodium channels, to the proximal compartment to inhibit neuronal action potentials, markedly decreased these strong contractions (supplementary material Movie 3). Out of nine innervated myotubes measured, six showed distinct strong contractions prior to TTX addition, all of which were abolished by TTX. By contrast, TTX in the neuronal compartment had no specific effect on the levels of weak, nearly static twitches in the same myotubes (Fig. 2E,F). To further address synapse functionality, we visualized cytoplasmic Ca^2+^ levels in myotubes and axons using Fluo-3AM. A rapid elevation of cytosolic Ca^2+^ levels is known to occur during the generation of muscular and neuronal action potentials. Ca^2+^ fluxes in the myotubes were correlated with both spontaneous and innervation-induced contractions (data not shown); however, we also observed Ca^2+^ flux events that were synchronized in axons and innervated myotubes, similar to the co-occurrence of innervated contraction described above. Non-innervated myotubes did not show correlated transients with other myotubes and axons (Fig. 3A,B; supplementary material Movie 4). To further validate that these synchronized Ca^2+^ transients represent bona fide motoneuron innervation-induced activity, we treated only the proximal neuronal compartment with TTX to block motoneuron firing. Whereas levels of spontaneous Ca^2+^ transients in non-innervated myotubes changed stochastically after TTX, transients in innervated myotubes were diminished in 90% of innervated myotubes observed (Fig. 3C,D), and synchronized neuromuscular transients were completely abolished. Taken together, these results demonstrate the formation of functional *in vitro* NMJs that enable direct stimulation of muscle electrical and contractile activity by motoneurons.

### Transmission of oxidative damage and pro-survival signaling between axonal and soma compartments

**Fig. 4.**
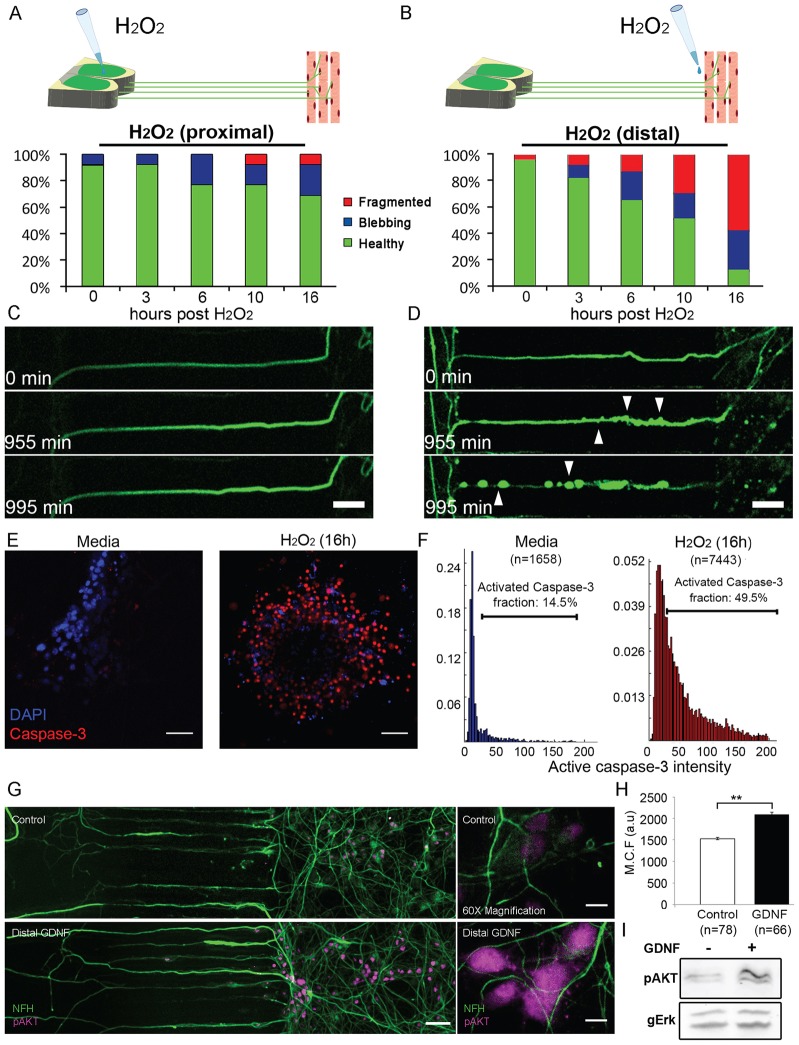
**Long distance progression of oxidative stress damage and GDNF-mediated pro-survival signaling.** (A,B) The graphs show the fraction of healthy, blebbing and fragmented axons after application of 1 mM H_2_O_2_ in (A) the neuronal/proximal or (B) muscle/distal compartment. (C,D) Timecourse images of HB9::GFP (green) axon degeneration. Images were taken with an epifluorescent microscope with a 10× objective. Arrowheads denote sites of axonal blebbing. Scale bars: 50 µm. (E) Epifluorescent, 20× objective images of immunostained spinal cord explants showing activated caspase-3 (red) and DAPI (blue) in the soma of control versus H_2_O_2_-treated spinal cord explants after 16 h. Scale bars: 100 µm. (F) Distribution of mean caspase-3 intensities in DAPI-positive ROIs. The caspase-3 intensity value of 40 was chosen as a cutoff between cell bodies with activated and inactivated caspase-3. (G) Stitched low-magnification image of the proximal, microgroove and distal areas of neurons in the microfluidic chamber, taken with a 20×objective. Images show phosphorylated Akt (pAkt)- and NFH-labeled neurons at 3 h after medium or GDNF treatment in the distal compartment. Scale bar: 50 µm. The right panel shows 60× objective images of neuronal soma in the proximal compartment. Scale bar: 10 µm. (H) Comparison of mean pAkt levels in cell bodies of GDNF-treated versus control-treated neurons. A total of ten fields per treatment were analyzed, containing 4–10 cells each. *n*, number of cells analyzed. Data show the mean±s.e.m.; **P<0.01 (one-tailed Student's *t*-test). (I) Representative western blot image of pAkt and gERK (total Erk1/2 as loading control) of lysate from the soma compartment of distal GDNF-treated versus medium-treated cultures.

Having established the presence of functional NMJs in the chamber, we sought to test functional aspects of NMJ biology utilizing the system. First, we tested differences in sensitivity to oxidative stress between the neuronal soma and NMJ compartments. When 1 mM H_2_O_2_ was added to the muscle compartment, disruption of distal axon contacts with myotubes was observed within minutes. Axonal degeneration started after 3–6 h at the distal compartment and spread backwards towards the neuronal cell bodies (Fig. 4B; supplementary material Movie 5). After 16 h, only 12% of the HB9::GFP axons recorded displayed a healthy intact morphology, whereas 30% and 58% were in blebbing and fragmented states, respectively (Fig. 4D) Lower H_2_O_2_ concentrations in the distal compartment result in milder degeneration; after 16 h at 100 µM, 79% of the axons remained intact while 21% were fragmented, whereas at 300 µM, 40% were healthy versus 10% and 50% that were blebbing and fragmented, respectively. To test the possibility that distal stress can activate signaling in the soma, we treated the NMJ compartment with 1 mM H_2_O_2_ and examined activation of the apoptotic effector protein caspase-3 in the soma and condensation of DNA in the nuclei (Fig. 4E). At 16 h after H_2_O_2_ application in the NMJ compartment, there was a threefold increase in the number of nuclei containing activated caspase-3 (Fig. 4F), consistent with a dying-back process. By contrast, addition of 1 mM H_2_O_2_ to the soma compartment did not result in visible NMJ or axon degeneration during a time frame of ∼18 h (Fig. 4A,C; supplementary material Movie 6). These data suggest that NMJs are more sensitive to H_2_O_2_ toxicity than the neuronal cell body, and that distal stress generates retrograde signals to the soma that activate apoptotic cell death pathways.

We additionally examined the role of GDNF, a neurotrophic factor known to promote motoneuron survival, taking advantage of the microfluidic culture to assess the spatial aspects of GDNF pro-survival signaling. Treatment of spinal cord neurons with GDNF in the distal compartment for 3 h resulted in an ∼40% elevation of phosphorylated Akt, a well-characterized pro-survival effector, in the cell bodies at the proximal compartment (Fig. 4G,H). By western blot analysis, we also observed a mean of 49% increase in phosphorylated Akt in the soma compartment (Fig. 4I). These results agree with those of Coulpier and Ibáñez, 2004, showing that GDNF applied to the distal compartment induces Akt phosphorylation that progresses in a retrograde manner to the soma. Thus, this microfluidic co-culture system can be used for studying spatial mechanisms of neurodegeneration versus survival at the subcellular level, and adding GDNF to the distal axons can activate survival pathways in the soma.

### GDNF enhances axon growth and innervation of myotubes when applied in the NMJ compartment

**Fig. 5.**
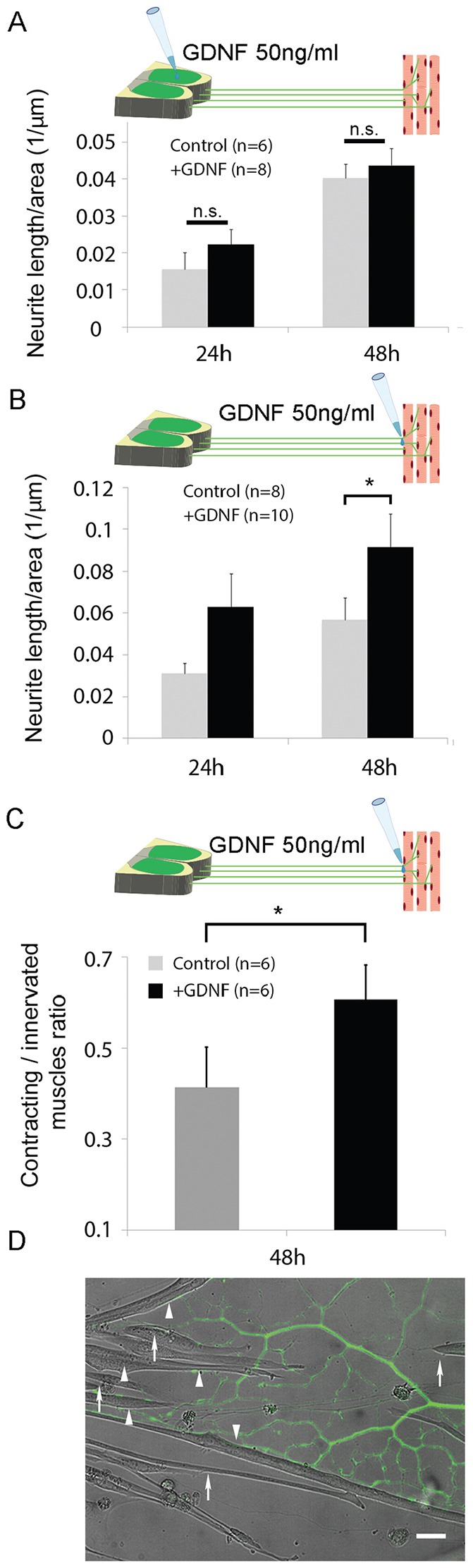
**GDNF facilitates axonal growth and muscle innervation when added specifically to the axonal/muscle compartment.** (A,B) Mean axonal length in the muscle compartment normalized to the area of measurement in the chamber treated with GDNF in (A) the neuronal cell body or (B) muscle compartments compared to the control. Two images of each co-culture muscle compartment from two independent experiments were pooled and analyzed. Data show the mean±s.e.m.; *P<0.05; n.s., non-significant (one-tailed Student's *t*-test). (C) Mean rate of functionally innervated muscles, measured according to their contractile behavior in the distal side GDNF treatment condition compared to the control, at 48 h after treatment. See Materials and Methods for further details. Data were taken from two independent experiments and show the mean±s.e.m.; *P<0.05 (one-tailed Student's *t*-test). (D) Representative epifluorescent and brightfield images, taken with a 20× objective, of myotubes innervated by HB9::GFP-expressing (green) axons that were not contracting (arrows) and myotubes displaying typical innervation-induced contraction (arrowheads). Scale bar: 25 µm.

Based on the observations that muscles attract axonal growth and the directed manner of innervation (Fig. 1A; Fig. 2A), we sought to use the MFC system to investigate how muscle-secreted factors participate in this process. We chose to focus on GDNF, as it is a known muscle-derived motoneuron survival factor as well as a promoter of NMJ activity (Wang et al., 2002b). However, little is known about its mechanism of action on motoneurons and NMJs, and in particular on the spatial aspects of its activity. To this end, we treated compartmental co-cultures with factor-deprived medium supplemented with GDNF in the muscle or neuronal soma compartments, and measured the extent of axon growth in the muscle compartment. We used co-cultures of 2–4 days *in vitro* (DIV), in which the extension of axons into the distal compartment had only initiated, and divided them between medium only and GDNF treatment. Compared with the control, GDNF in the NMJ compartment increased the growth of axons in the distal compartment after 24 and 48 h by twofold and 60%, respectively, although only after 48 h did the effect reach statistical significance (Fig. 5B). Surprisingly, despite the previous observation of activation of survival pathways in the soma upon addition of GDNF to the distal side, no axonal-growth-promoting effect was observed for GDNF added to the soma compartment (Fig. 5A), demonstrating spatially distinct mechanisms for these two GDNF-dependent processes.

We then examined spatial aspects of GDNF application on the formation of functional NMJs. At 48 h after GDNF treatment, we measured the fraction of myotubes colocalized with an axon that also displayed innervation-specific contraction in each compartment (Fig. 5D, see Materials and Methods for details on the measurement technique). In agreement with the axonal growth results, GDNF application to the distal compartment increased the fraction of actively innervated myotubes by ∼50% in comparison to controls (0.62 versus 0.41, Fig. 5C), whereas treatment in the soma had no effect (0.44 versus 0.42, data not shown). Overall, these results suggest that GDNF has different effects on motoneuron axonal outgrowth and NMJ formation, depending on its site of activity.

### GDNF secreted from muscles undergoes retrograde transport in motoneurons

**Fig. 6.**
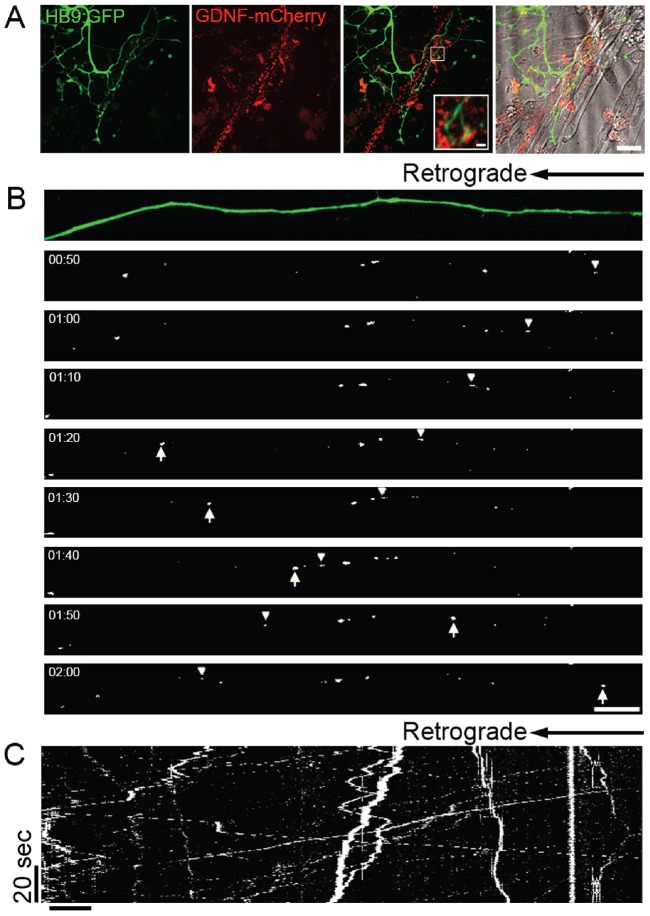
**Retrograde transport of muscle-secreted GDNF in motoneuron axons.** (A) Confocal 60× objective images of HB9::GFP-expressing axons (green) in contact with myocytes expressing GDNF–mCherry (red) at 5 days after lentiviral infection. (B) Timecourse images taken using a confocal microscope and a 60× objective showing retrograde (arrowheads) and anterograde (arrows) transport of GDNF–mCherry in a HB9::GFP-expressing motoneuron. (C) Kymograph of GDNF–mCherry transport along an HB9::GFP-expressing axon. Scale bars: 20 µm (A), 10 µm (B,C), 2 µm (A, inset).

Although we did not observe effects of GDNF on axonal growth and innervation when applied to the soma, pro-survival signaling was activated when GDNF was applied to the distal compartment (Fig. 4G–I). This suggests that survival signals can be mediated by long-distance retrograde axonal transport of GDNF secreted from the muscle, as in neurotrophin signaling endosomes (Harrington and Ginty, 2013; Ibáñez, 2007). In order to follow the retrograde muscle-to-neuron uptake and transport of GDNF, we infected myoblasts in the MFC with lentivirus (LV) vectors for GDNF–mCherry expression (Fig. 6A), and tracked the transport of mCherry-positive puncta along innervating axons (Fig. 6B). Importantly, GDNF–mCherry expression could not be detected in the spinal cord explant at 5 days after infection of myocytes (data not shown), indicating that virions could not diffuse to the motoneuron cell body compartment and did not infect motoneurons. Moreover, no GDNF transport was seen in axons that did not reach the muscle compartment and thus had no contact with muscles (data not shown), suggesting that GDNF uptake by axons occurs at sites of high GDNF concentration, such as NMJs. After 5 DIV, ∼75% of transported GDNF particles moved primarily in the retrograde direction (mean±s.e.m.; instantaneous velocity of 1.36±0.06 µm/s, *n* = 170 events). A smaller number of particles, however, were found to move in the anterograde direction, at least partially (mean±s.e.m.; velocity of 1.96±0.16 µm/s, *n* = 99 events, Fig. 6B,C; supplementary material Movie 7). As GDNF–mCherry in this experiment can only be derived from the distal muscle, the observed anterograde movement could result from particles with a net retrograde directionality switching to the anterograde direction. Thus, the microfluidic co-culture system can be used to study bidirectional signaling between motoneurons and muscles mediated by secreted factors like GDNF. Furthermore, we demonstrate directly that GDNF is secreted from the muscle to the neuron and undergoes processive transport along the axon.

## DISCUSSION

Here, we have described a compartmental microfluidic chamber system for visualizing and manipulating NMJs. Using both staining and functional assays, we demonstrate the formation of NMJs from primary muscle cultures and spinal motor neurons. The interaction between axon terminals and muscle cells forms functional NMJs, as shown before in mass co-cultures (Daniels et al., 2000; Dutton et al., 1995; Giller et al., 1973; Nelson et al., 1993). However, unlike mass co-cultures, compartmentalized microfluidic culture platforms distinguish local versus distal signaling mechanisms and allow accurate monitoring of antero- and retrograde axonal transport as well as the localized manipulation of signaling pathways that regulate NMJ formation and/or maintenance and cell survival. Furthermore, this method allows spatial and temporal control over microenvironments by manipulating either neuron or muscle cell populations independently, allowing investigation of mechanisms of cell–cell communication and NMJ formation and maintenance. Unlike other co-culture systems that were published recently (Park et al., 2013; Southam et al., 2013), here, we use HB9::GFP explants and not motoneuron cultures. This has a number of advantages, including easier and more accurate dissection of the motor area, facilitation of motoneuron imaging, increasing the probability of axons penetrating the grooves, decreasing chamber variability and overall providing a model system that closely mimics the *in vivo* situation.

**Fig. 7.**
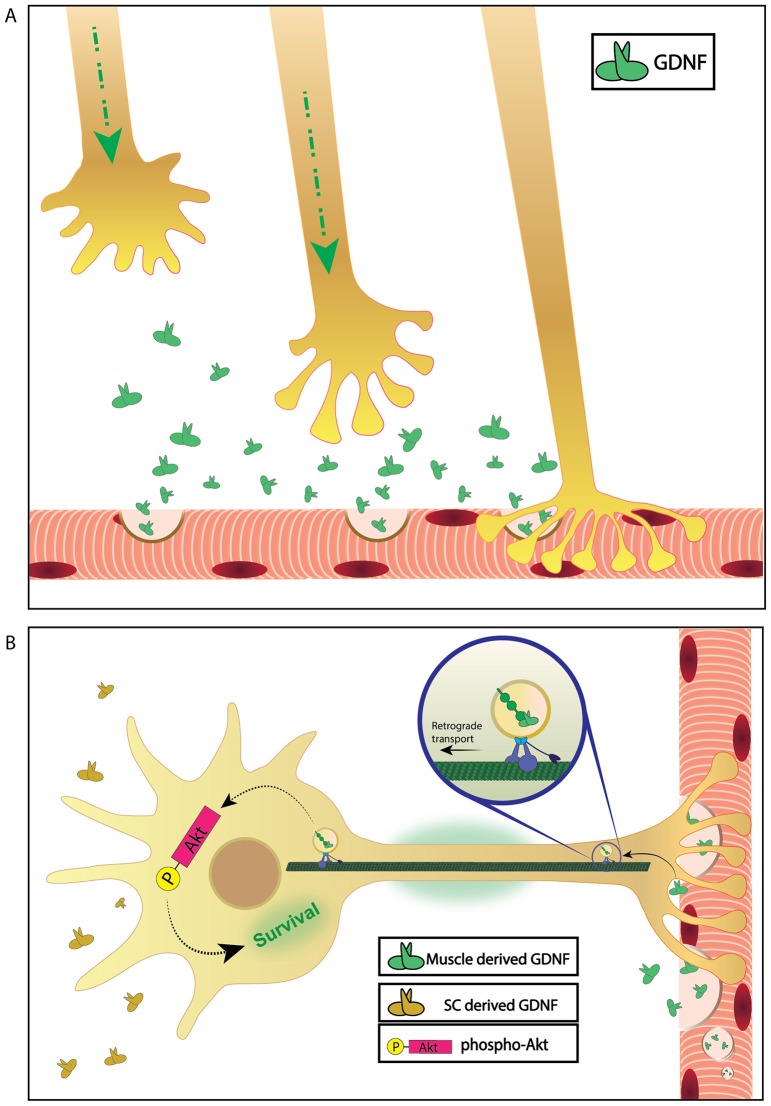
**Model depicting the dual function of GDNF in motoneurons in promoting local axonal growth and innervation and long-distance survival signaling.** (A) Local effect of GDNF on axonal growth, guidance and NMJ formation. (B) Long-distance effect of retrograde-transported GDNF in activating pro-survival signals in the cell body.

We used the microfluidics co-culture system to examine spatially distinct aspects of GDNF functions at the cell soma and the distal axons. We demonstrated that GDNF promotes axonal growth and innervation of muscles only upon local application, whereas survival signaling through Akt is activated in the soma. These findings suggest spatial specification of GDNF effects, facilitating growth and muscle innervation at axon tips and survival pathways in the soma (Fig. 7).

Interestingly, we show that axons at the NMJ compartment are more vulnerable to local H_2_O_2_ stress than the neuronal cell bodies and proximal neurites. NMJ disruption ensues shortly after H_2_O_2_ treatment, and the retrograde progression of the degeneration provides a direct demonstration of a dying-back mechanism. This dying-back degeneration process occurs when low concentrations of H_2_O_2_ are applied to the NMJ, but not the cell body compartment. The reasons for increased vulnerability of the synapses will be an intriguing topic for future work. This might be due to an intrinsically higher buffering capacity in the soma or signaling pathways that improve the cell body response to stress compared with that of the distal processes of the axon. Another possibility is that stress signals from the muscle cells cause NMJ disruption, thus initiating the degeneration cascade. Likewise, supporting cells originating from the spinal cord might protect motor neurons at the proximal compartment.

Furthermore, using this platform enables the elucidation of one of the most fundamental questions regarding the mechanisms of neurodegeneration: where does it all begin (Conforti et al., 2007)? Is neurodegeneration a distal process beginning with synapse disruption followed by a dying-back mechanism (Dadon-Nachum et al., 2011; Fischer et al., 2004; Gould et al., 2006; Krakora et al., 2012) or is it a general cell-wide death event (Kole et al., 2013; Pasinelli et al., 1998; Sathasivam et al., 2001)? Our data with H_2_O_2_ toxicity suggest that inducing stress results first in a disruption of the distal compartment followed by a dying-back mechanism. Future studies using this platform with specific pathological models could shed light on the molecular triggers of diverse neurodegenerative diseases as well as nerve injury. This compartmentalized system enables distinction between dying-back and dying-forward mechanisms. Future studies with more defined temporal resolution will provide further details on initiation and progression of neurodegenerative events.

A key finding in this study is the characterization of spatial specificity in GDNF effects, and the visualization of GDNF trafficking from muscle to neuron. GDNF has a potential therapeutic effect in different pathological paradigms, such as Parkinson's disease, brain ischemia, epilepsy and amyotrophic lateral sclerosis (ALS) (Acsadi et al., 2002; Airaksinen and Saarma, 2002; Krakora et al., 2013; Mohajeri et al., 1999; Moreno-Igoa et al., 2012). Quantitative analysis of GDNF expression in skeletal muscle revealed its presence during postnatal development and into adulthood, further suggesting a role in both NMJ development and maintenance (Nagano and Suzuki, 2003). Indeed, in *Xenopus*, GDNF treatment of nerve–muscle co-cultures facilitates NMJ development (Wang et al., 2002a). Therefore, understanding the spatial constraints and specificity of GDNF effects are of keen interest. Interestingly, it was previously shown in PC12 cells that nerve growth factor (NGF) exerts its pro-survival effect through activation of its TrkA (also known as NTRK1) receptor on the cell surface, whereas the neurite extension effect requires receptor internalization (Zhang et al., 2000). However, in our study, GDNF has a different effect – at the distal side it facilitates axon growth and in the soma it signals for cell survival. This is in agreement with a previous study showing retrograde-only transport of survival signaling as well as neurite extension upon distal application of GDNF in sympathetic neurons (Coulpier and Ibáñez, 2004). Possibly, these divergent effects are due to a difference between the two ligand–receptor signaling mechanisms or due to the different cell types examined. Indeed, and in agreement with what we found here, the conditional ablation of the GDNF receptor RET in motoneurons leads to a reduced number of NMJs and severe disruption of pre-synaptic terminals (Baudet et al., 2008), suggesting a local GDNF effect. Motoneuron survival in the spinal cord was also affected in that study, implying an unknown mode of crosstalk with the long-distance survival pathways. Moreover, increasing GDNF levels in mouse muscle enhances multiple innervation and delays synapse elimination (Keller-Peck et al., 2001; Nguyen et al., 1998). Interestingly, however, overexpression of GDNF in the spinal cord did not cause hyper-innervation of the muscle, suggesting the importance of a specific post-synaptic origin of GDNF (Nguyen et al., 1998). GDNF might also have a role in post-synaptic organization by directly affecting the muscles, as it was previously suggested to promote the insertion of AChRs into the surface membrane (Yang and Nelson, 2004). The microfluidics platform that we present here will allow manipulation of GDNF receptors and co-receptors to study its mechanism of action and answer key questions regarding its specific spatial activity. As GDNF has several receptors (Sariola and Saarma, 2003), it is tempting to postulate that specific receptor complexes are required for survival versus growth and innervation pathways. This will be an interesting avenue for future work.

To summarize, we have developed a powerful *in vitro* platform for NMJ studies, consisting of a microfluidics compartmentalized culture with motoneuron cell bodies in one compartment and muscle cells in the other. This platform provides a simplified environment for studying the mechanisms of NMJ formation, maintenance and disruption. We verified that functional NMJs are formed within the microfluidic chamber and demonstrated the utility of this system to investigate mechanisms of GDNF spatial function, signaling and transport.

## MATERIALS AND METHODS

### Animals

HB9::GFP mice were originally obtained from Jackson Laboratories, and the colony was maintained by breeding with ICR mice. All animal experimentation was approved by the Animal Ethics Committee of Tel Aviv University. Primary cultures and spinal cord explants were taken from mice of either sex.

### Skeletal myocyte culture

Skeletal muscle cultures were derived from the gastrocnemius muscle of adult mice (1–4 months old) using techniques modified from previous studies (Beauchamp et al., 2000). Briefly, the skin was removed from hind limbs and the gastrocnemius muscle was dissected from bones and incubated in 2 mg/ml collagenase I (C0130, Sigma) in DMEM containing 2.5% penicillin-streptomycin-nystatin (PSN) for 3 h. Myofibers were then triturated and incubated for 3 days in Matrigel-coated (FAL356234, BD Biosciences) six-well plates with Bioamf-2 medium (01-194-1A, Biological Industries) with 1% PSN at a density of 100–120 myofibers per well. For enrichment of the myoblast population, adherent cells were trypsinized and pre-plated in an uncoated dish for 1 h at 37°C. Non-adherent cells were then transferred into a Matrigel-coated dish with Bioamf-2 medium. Pre-plating was performed for two consecutive days, keeping the culture at less than 50% confluence, before plating cells in the chamber. Cultures were maintained in 37°C and 5% CO_2_.

### Spinal cord explant

Spinal cords were dissected from embryonic day 11–12 HB9::GFP mice and stripped of meninges and dorsal root ganglia. The ventral horn was separated from the dorsal horn by longitudinal cuts along the spinal cord, and transverse sections up to 1 mm were placed in the explant well.

### Microfluidic chamber preparation

Polydimethylsilxane (PDMS) microfluidic chambers (MFC) were designed and cast as described previously (Gluska et al., 2014; Park et al., 2006). After punching the wells, a small ‘cave’ was made in the explant well near the grooves using a 25G needle to keep the explant in place. Microfluidic devices were cleaned of surface particles using adhesive tape and were sterilized in 70% ethanol for 15 min. Devices were dried completely under sterile conditions including UV radiation, attached to a sterile 60-mm plastic dish (Nunc) using gentle pressure, and margins were sealed with PDMS before incubation at 60°C for 30 min to prevent detachment of the chamber. Muscle channels were coated with Matrigel diluted 1∶10 with DMEM containing 2.5% PSN for 30 min at 37°C, before filling the muscle wells with 150 µl of Bioamf-2 medium. The explant well and channel were filled with 150 µl of 1.5 ng/ml polyornithine (P-8638, Sigma) in PBS overnight, and then replaced with 150 µl laminin (L-2020, Sigma), 1∶333 in deionized distilled water (DDW) overnight. At 1 day before plating the spinal cord explant, laminin was replaced with explant medium [Neurobasal (Life Technologies) supplemented with 2% B27 (Invitrogen), 1% penicillin-streptomycin (Biological Industries), 1% Glutamax (Life Technologies), 25 ng/ml brain-derived neurotrophic factor (Alomone Labs)] until the day on which co-culturing was started.

### Assembly and maintenance of cultures in chambers

On the day of plating, medium was removed from the muscle compartment, 20,000 myoblasts in 300 µl of Bioamf-2 containing 1% PSN were plated in the muscle compartment (150 µl per well). After 3 days, medium was removed from all compartments and the spinal cord explant was plated in the explant cave in 4 µl of HBSS containing 1% penicillin-streptomycin, and incubated for 1 h at 37°C. Explant and muscle medium (Bioamf-2) were added to the neuron and muscle wells, respectively. Explants were then examined under a microscope and, if needed, were placed back into the optimal location using a pipette. At 1 day after explant plating, spinal cord medium was replaced and supplemented with 10 µM Ara-C to prevent fibroblast and glial proliferation and migration into the muscle compartment. Cultures were examined daily and following the initial crossing of axons to the distal compartment, media in both compartments were replaced with poor neurobasal (PNB, neurobasal medium supplemented with 1% Glutamax, 1% penicillin-streptomycin). Cultures were maintained at 37°C and 5% CO_2_, and medium was refreshed every 2–3 days.

### Minimized MFC for dispersed motoneuron culture

For enriched motoneuron cultures, silicon microfluidic chambers were fabricated as described above using a minimized mold (channel width and length of 750 µm and 8 mm, respectively; microgroove width and length of 15 µm and 300 µm, respectively). 7-mm wells were punched at the both sides of each channel. Chambers were then attached to glass-bottomed dishes and coated with 0.001% poly-L-lysine (Sigma, P4832) and laminin in pure H_2_O overnight at room temperature. This was replaced with neurobasal medium and chambers were maintained at 37°C, 5% CO_2_ until plating the neurons.

### Immunofluorescence

Cultures were fixed in 4% paraformaldehyde and permeabilized with 0.1% Triton X-100 in PBS. Primary antibodies against NFH (Abcam, ab72996; 1∶1000), synapsin-I (Millipore, AB1543P; 1∶100), activated caspase-3 (BioVision, 3015–100; 1∶100) and phosphorylated (S473) Akt (Abcam ab66138; 1∶200) were diluted in blocking solution (5% donkey serum, 1 mg/ml BSA IgG and protease free, in PBS) and incubated overnight at 4°C. Samples were incubated with species-specific secondary antibodies (Jackson Immunoresearch) for 2 h at room temperature. TMR-conjugated α-bungarotoxin (Sigma, T0195) staining for evidence of acetylcholine receptor (AChR) clusters was performed at 1 µg/ml in PBS for 15 min prior to cell permeabilization and primary antibody incubation. For visualization of nuclei in myotubes, Hoechst 33258 (1 µg/ml) or DAPI was used for live or fixed samples, respectively. After the staining protocol was completed, MFC was peeled from the dish by gently pulling it from the proximal to distal side, to minimize severing of axons. Prolong mounting medium was added and covered with a #1.5, 18×18 mm coverslide.

### Fluorescence microscopy and image analysis

Unless otherwise stated, all confocal, epifluorescence and brightfield images were captured using a Nikon Ti microscope equipped with Yokogawa CSU X-1 spinning disc, controlled by Andor IQ2 software. Epifluorescence was imaged using the same microscope in widefield mode. Confocal and widefield images were captured with Andor iXon897 EMCCD and Neo sCMOS cameras, respectively. In all live-imaging assays performed, imaging of myotube contractions, Fluo-3 transients, FM dye, axonal degeneration after H_2_O_2_ and axonal transport, living samples were maintained in a humidified incubation chamber at 37°C, 5% CO_2_. Details of fluorescent labels and excitation and emission parameters are given in supplementary material Table S1 below. Images were analyzed using ImageJ software, except for BTX-HB9::GFP colocalization, which was analyzed using Imaris (Bitplane) Coloc module and 3D rendering and surface modeling, which were performed using Imaris Surpass and Surface functions.

### Analysis of colocalization of BTX and motoneurons

To visualize putative NMJ sites, α-bungarotoxin was added for 15 min to a 5 DIV co-culture chamber with HB9::GFP motoneurons and myotubes. Live images were taken using a confocal microscope, and colocalization analysis was performed using Imaris software (Bitplane). Random colocalization was assessed in the same pictures by flipping the GFP channel horizontally against the BTX.

### FM dye uptake and release assay

For visualization of pre-synaptic sites, medium was removed from the chamber, and 15 µM FM-4-64 dye (Life Technologies) was added in depolarization solution (31.5 mM NaCl, 90 mM KCl, 5 mM HEPES, 1 mM MgCl_2_, 2 mM CaCl_2_, 30 mM glucose) for 2 min at 37°C. FM dye was washed twice for 5 min at 37°C with FM wash buffer (DMEM without Phenol Red, supplemented with 1% Glutamax, 50 µM DL-APV, 10 µM DNQX). Samples were promptly imaged under a confocal microscope for uptake levels of FM dye. Release images were taken 2–5 min after replacing the proximal medium with stimulation solution (depolarization solution supplemented with 10 µM glutamate).

### Measurement of innervation-induced contraction in myotubes

Chambers of co-cultures at 7 DIV were examined by live microscopy. For characterization of contractile behavior, denervated and HB9::GFP innervated myotubes were recorded using brightfield at 30 frames per second (fps) for 66 s, before and after 1 µM TTX was added to the neuronal compartment. Time-lapse images were taken for analysis using ImageJ. To create a time trace of contractions, high contrast (bright or dark) regions of interest (ROIs) were selected on each myotube. Movement of the selected spot due to myotube contraction was assessed by the change in the ROI intensity over time. Intensities were normalized to time 0 and to the average intensity along the trace, to enhance transient changes in the signal. Irregular high-amplitude peak representations of strong myotube contractions were typical in innervated myotubes, whereas small frequent peaks represented weak vibration-like contractions, common in both innervated and denervated myotubes. The number of strong contractions, as measured from the time trace, was manually validated by re-examining the time-lapse image series. The number of strong and weak contractions in innervated myotubes was compared before and after TTX was added to the neuronal compartment. A myotube with a post to pre TTX difference of >50% was measured as an increase or decrease in contraction, and the fraction of increased, decreased and unchanged myotubes was calculated. For measurement of the GDNF effect on NMJ formation, myotubes contacted by neurons (i.e. innervated myotubes) were examined by high-speed live microscopy for at least 1 min and counted as actively innervated if they displayed strong and irregular contractions or co-contraction with two or more innervated myotubes in the same field of view. Then, the fraction of actively innervated myotubes in each co-culture was calculated.

### Cellular Ca^2+^ imaging using Fluo-3AM

Fluo-3AM (Biotium) 1 mM in DMSO was diluted to 2 µM in neurobasal medium and placed in the muscle compartment of 7 DIV co-cultures. After 20 min of incubation at 37°C, the MFC was placed in the confocal microscope and fluorescence from innervated and denervated myotubes was recorded at 30 fps for 30–60 s, before and after 1 µM TTX addition to the neuronal compartment. Intensity time-traces of axons and myotubes was extracted using ImageJ, and plotted using Matlab software (Mathworks). Spikes in the Fluo-3AM signal were manually counted and compared after TTX between innervated and denervated myotubes, similar to comparison of contractile behavior as described above.

### Oxidative stress at the distal or proximal side of the chamber

To induce oxidative stress, specific concentrations of H_2_O_2_ were added to the distal or proximal side of the chamber, while keeping the opposite channel with higher medium volume to avoid leakage of H_2_O_2_ to the other side. Live imaging of 5-min intervals over several hours was performed using confocal microscopy. To assess progression of neuronal damage over time, axons were divided into three groups: healthy, blebbing (showing early or minor degeneration processes but still intact) or fragmented, where severe or complete fragmentation was observed. To measure the difference in neuronal soma caspase-3 activation after oxidative stress in the distal compartment, confocal *z*-stacks of DAPI and active-caspase-3-stained spinal cord explants were processed using ImageJ. First, a threshold of the DAPI channel was set to create ROIs of the cell nuclei, and mean caspase-3 signal was calculated in those ROIs. Then, resulting intensity values were plotted as a histogram using Matlab, and a threshold value was manually selected to compare the fraction of caspase-3-activated cell bodies between 1 mM H_2_O_2_ and medium-treated NMJ compartments.

### Akt phosphorylation

For Akt phosphorylation analysis, enriched motoneurons were cultured as described previously (Henderson and Bloch-Gallego, 1995), and plated in the proximal side of a minimized MFC described above. At 4 DIV, medium in the proximal cell was replaced with neurobasal medium or neurobasal medium supplemented with 100 ng/ml GDNF (Alomone labs). After 3 h, chambers were taken for either immunofluorescence or western blot analysis. For immunofluorescence, samples were fixed, permeabilized and stained with NFH and pAkt antibodies. Imaging was performed using 20× and 60× objectives in the confocal microscope. Maximum intensity projections of 15 slices per field were created using ImageJ. To show axonal crossing from the proximal to distal sides, adjacent images were stitched together (Fig. 4G). For quantification of pAkt, cell areas were traced and saved as ROIs, and the mean pAkt intensity (total pixel value/area) was measured. For western blotting, proximal sides of the samples were lysed in sample buffer, using a minimal volume (50–70 µl in total). To prevent contamination by axons in the distal side, an excess of PBS was added in that compartment. Following lysis, samples were run on 10% SDS-PAGE gels, blotted onto nitrocellulose membranes, blocked in TBST with 5% skim milk for 1 h, then probed using antibodies against pAkt (Abcam, ab66138; 1∶400) and ERK1/2 (Sigma, M5670; 1∶15,000) for 2 h. After secondary antibody incubation and ECL exposure, blots were quantified using the ImageJ gel analysis function.

### Treatment with GDNF for measurement of axon growth and muscle innervation

Co-cultures of 3–5 DIV with initial crossing of axons into the distal compartment were selected for experiments on GDNF effects on axon length and NMJ activity. In each experiment, two to four co-cultures were allocated to each condition. In GDNF-treated cultures, medium was replaced with poor neurobasal medium supplemented with 50 ng/ml GDNF (Alomone labs) in the appropriate compartment.

### Measurement of axon growth

Brightfield images were acquired using the FLoid® Cell Imaging Station (Life Technologies), over 2 days. Two random fields in the distal compartment across from the explant were imaged. Neurites were manually traced, saved as ROIs and measured using ImageJ, as was the relevant area in each field. Neurite length was calculated as the sum of all neurites normalized to the imaged field area.

### Lentivirus GDNF–mCherry production

The GDNF plasmid was a kind gift from Mart Saarma from the University of Helsinki. The pmCherry-N1 vector was a kind gift from the laboratory of Koret Hirschberg, Tel-Aviv University. GDNF and mCherry were subcloned into a viral vector (pLL-mCherry-GDNF). The helper plasmids pVSVG and pGag-Pol were a kind gift from Eran Bacharach, Tel-Aviv University. Lentiviruses bearing the sequence encoding mCherry–GDNF were generated by transfecting subconfluent 293T cells in 100-mm plates with 20 µg of pLL-mCherry-GDNF together with 15 µg of pGag-Pol and 5 µg of pVSVG using the calcium phosphate procedure. Culture supernatants were harvested at 2 days post-transfection, and concentrated ×100 using the PEG virus precipitation kit (Abcam, ab102538).

### Muscle infection and GDNF transport in axons

Myoblasts and explants were plated in chambers and incubated for 1 h, after which 14 µl of concentrated lentiviral particles encoding mCherry–GDNF were added to the muscle compartment. A volume difference between the compartments was created in order to prevent viral particles from diffusing to the explant compartment. GDNF transport in HB9::GFP axons was observed at 5 days post infection by live imaging using confocal microscopy. Time-lapse series were transformed to kymographs using ImageJ. Transport velocities were obtained by manually tracking GDNF puncta using the manual tracking plugin.

## Supplementary Material

Supplementary Material
