# Supplementary material for: A compartmentalized microfluidic neuromuscular co-culture system reveals spatial aspects of GDNF functions
Source: J Cell Sci. 2015 Mar 15;128(6):1241–52. doi: 10.1242/jcs.167544 (PMC4359927; doi:10.1242/jcs.167544)
Supplement: Supplementary Material [file supp_128_6_1241__index.html]

A compartmentalized microfluidic neuromuscular co-culture system reveals spatial aspects of GDNF functions — Supplementary Material 

# A compartmentalized microfluidic neuromuscular co-culture system reveals spatial aspects of GDNF functions

## JCS167544 Supplementary Material

**Files in this Data Supplement:**

- **Supplementary Material**
