# Supplementary material for: A compartmentalized microfluidic neuromuscular co-culture system reveals spatial aspects of GDNF functions
Source: J Cell Sci. 2015 Mar 15;128(6):1241–52. doi: 10.1242/jcs.167544 (PMC4359927; doi:10.1242/jcs.167544)
Supplement: Supplementary Material [file supp_128.6.1241_JCS167544.pdf]

Supplementary Figure Legend:

Fig. S1. Scheme of neuromuscular co-culture system. (A) Layout of dissection, culturing and plating of embryonic spinal cord explants and myocytes (upper & lower panels, respectively) in the microfluidic chamber. (B) Timeline of co-cultures and assays.

Supplementary movies:

Movie 1. Volume rendered image of motoneuron endplate contacting myotube AChR cluster. Live confocal image stack taken with 150X magnification of HB9::GFP labeled motoneuron (green) and BTX-594 stained AChR on the myotube (red). Volume rendering (left) and 3D surface model (right) were made using Imaris software.

Movie 2. Synchronous contraction of myotube innervated by MN. HB9::GFP neurons (in green) innervate numerous cultured myotubes. Samples were imaged at 33 fps. Synchronous contraction of several innervated in denoted by arrows.

Movie 3. TTX in the proximal neuronal compartment abolishes strong contractions in innervated myotubes. HB9::GFP neurons (in green) innervate a cultured myotube. Samples were imaged at 40 fps. Image series were taken before and 15min after addition of 1μM TTX.

Movie 4. Correlated Fluo-3 signal spike in myotubes and innervating axon. Fluorescence signal of Fluo-3 was imaged by confocal microscopy at 33 fps. Arrows and arrowheads point to innervated myotubes and innervating axon, respectively. Please note that the absolute signal increase in myotubes is much higher than in the axon, although the fold difference in the neuron reaches comparable levels (see Figure 3G).

Movie 5. Axon degeneration after addition of 1 mM H_2O_2 to the NMJ compartment. 1 mM H_2O_2 was added to the NMJ side of 9 DIV co-culture chamber.

HB9::GFP MNs in chamber were imaged every 5 min over several hours using spinning disc confocal microscopy.

Movie 6. Axon degeneration after addition of 1 mM H_2O_2 to the cell body compartment. 1 mM H_2O_2 was added to the cell body side of 9 DIV co-culture chamber. HB9::GFP MNs were imaged every 5 min over several hours, using spinning disc confocal microscopy.

Movie 7. Retrograde GDNF transport along axons. Myocytes were infected with GDNF-mCherry and time lapse movies of innervated axons were taken 5 days later. Anterograde motility is directed toward the left and retrograde motility is directed toward the right. Images were acquired at 1 frame/s using a spinning disc confocal microscope.

Supplementary figure 1

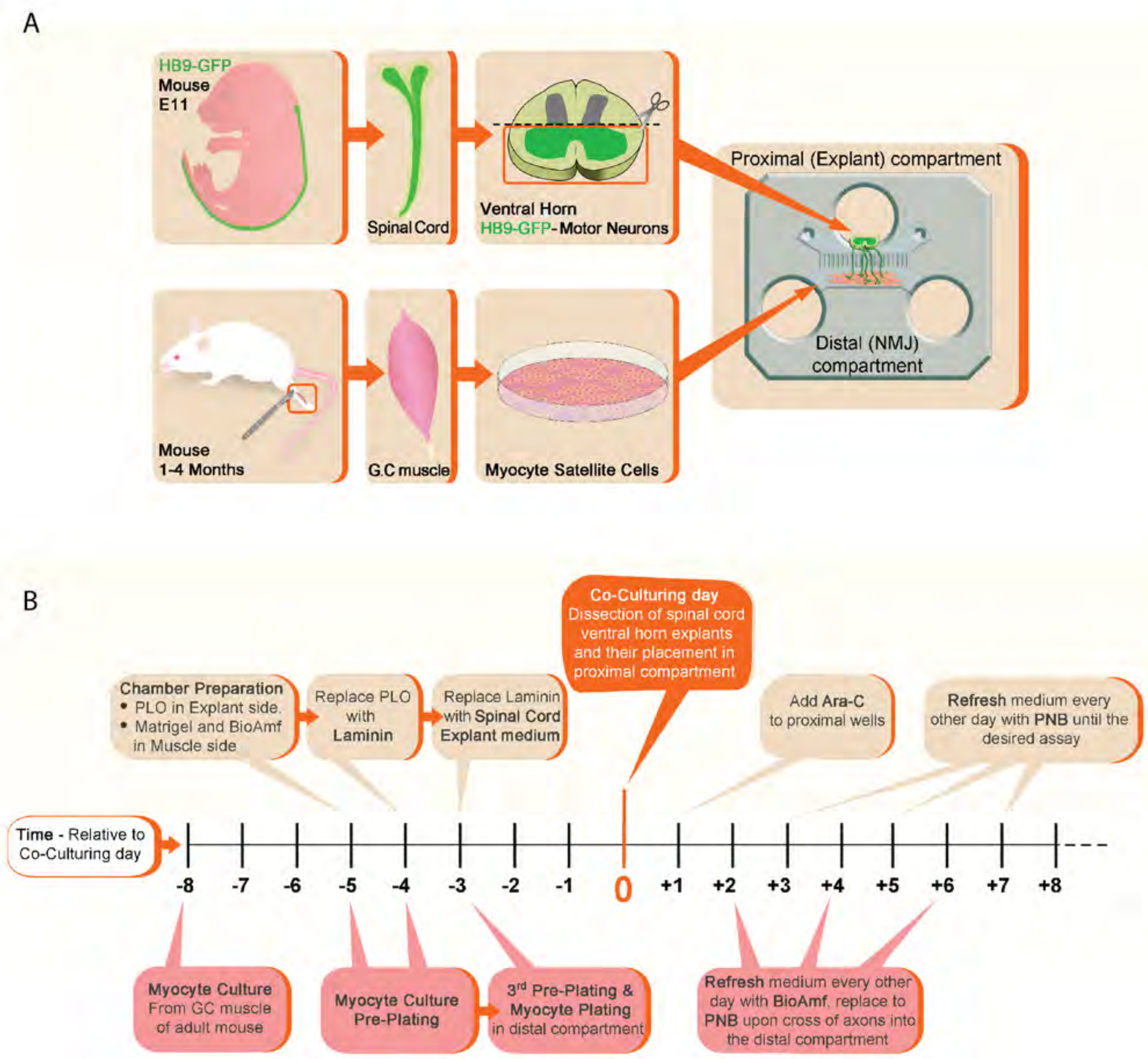

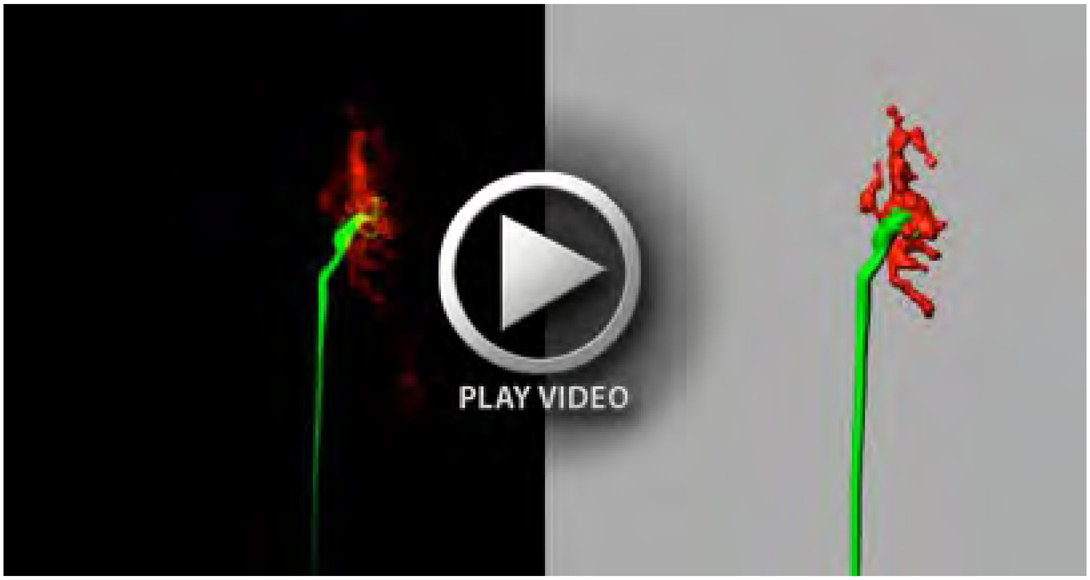

Movie 1.

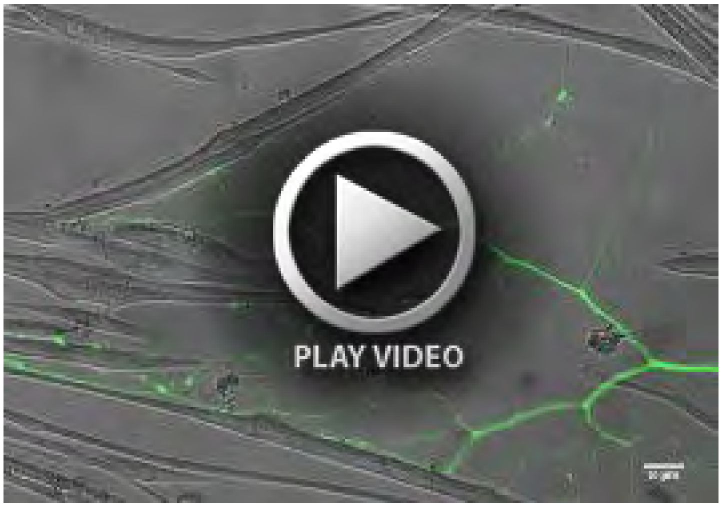

Movie 2.

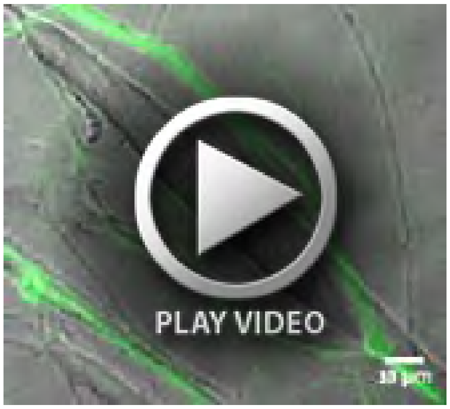

Movie 3.

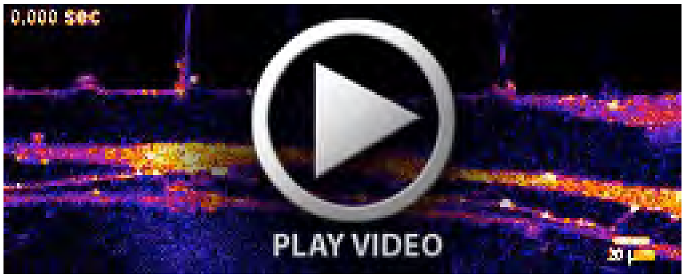

Movie 4.

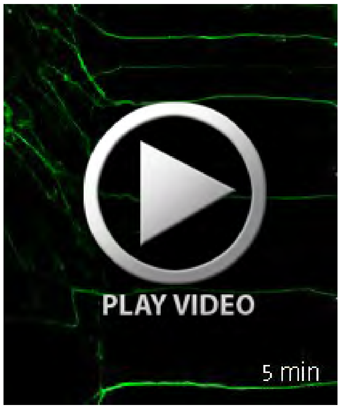

Movie 5.

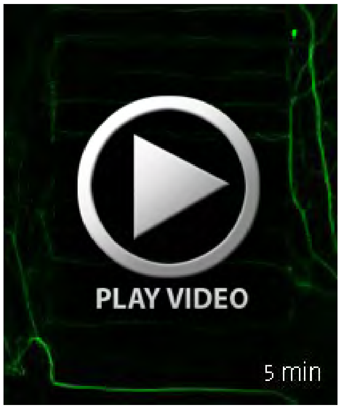

Movie 6.

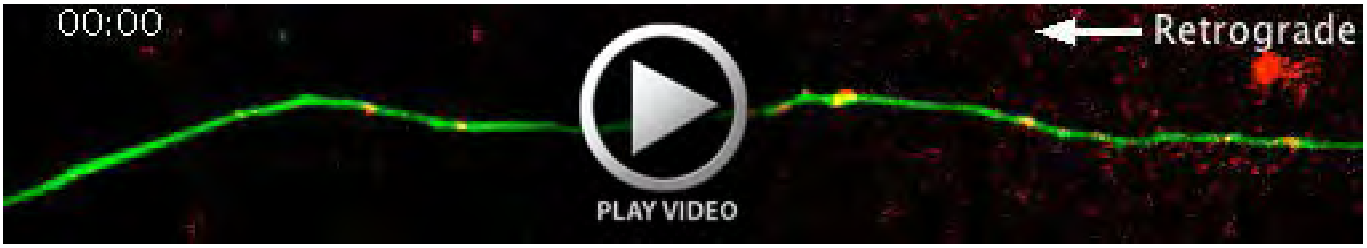

Movie 7.
